# Segmental Testicular Infarction

**DOI:** 10.5334/jbsr.4217

**Published:** 2026-03-10

**Authors:** Harmonie Candelier, Vassiliki Pasoglou, Sandy Van Nieuwenhove

**Affiliations:** 1 Department of Radiology, Cliniques Universitaires de Saint Luc, Université Catholique de Louvain, Brussels, Belgium

**Keywords:** testicular hematoma, scrotal ultrasound, magnetic resonance imaging

## Abstract

*Teaching point*: Segmental testicular infarction is a rare entity that remains poorly documented in the literature. Awareness of its imaging evolution is essential to avoid unnecessary surgical intervention.

## Clinical History

**Figure 1:**
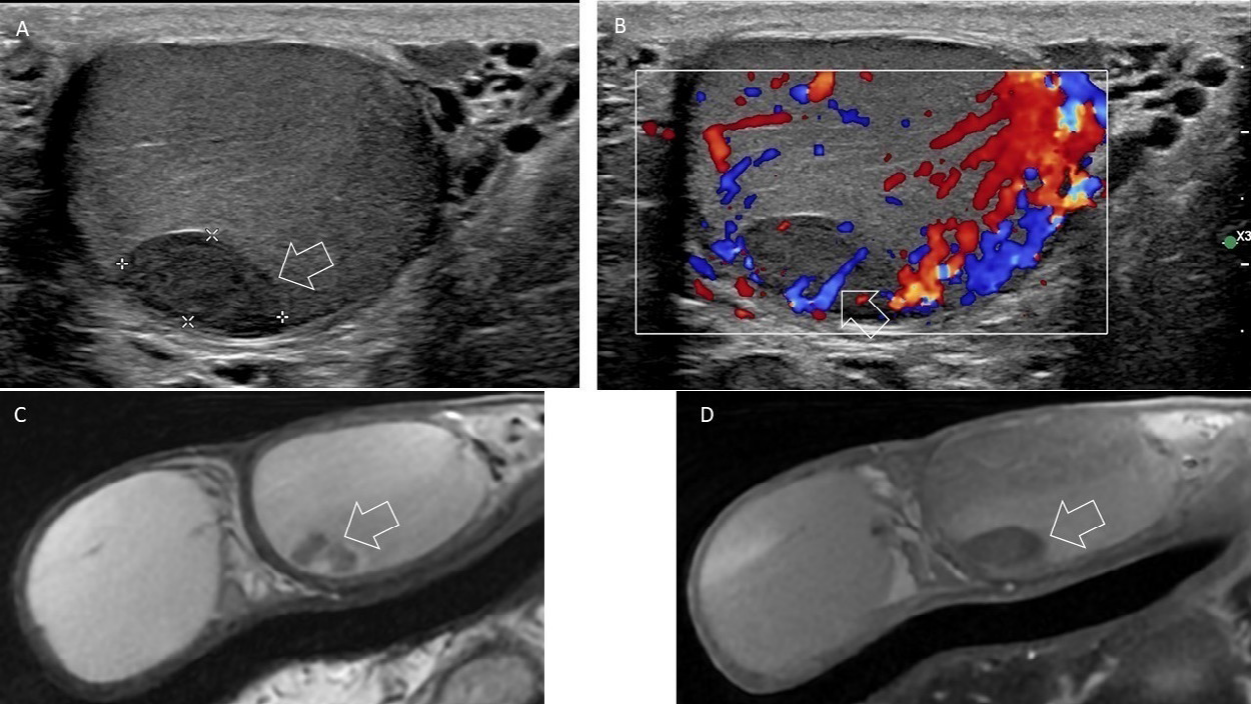
**(A)** Scrotal ultrasound showed a well‑defined hypoechoic lesion (arrow) **(B)** Color Doppler ultrasound showed no vascular distortion (arrow). **(C)** Axial T2‑weighted images showed a nodular hypointense lesion with hyperintense rim (arrow). **(D)** Post‑contrast T1‑weighted images showed no enhancement (arrow).

A 28‑year‑old patient presented to the emergency department with acute‑onset left testicular pain. There was no history of trauma, infection, or known coagulation disorder. Scrotal ultrasonography demonstrated a well‑defined, subscapular, hypoechoic focal lesion measuring 13 × 7 mm (Figure 1A). Color Doppler ultrasound showed no vascular distortion (Figure 1B).

Given the indeterminate nature of the lesion and the significant anxiety expressed by the patient, scrotal MRI was performed. MRI revealed a nodular lesion with predominant low signal intensity on T2 surrounded by a peripheral hyperintense rim (Figure 1C). No enhancement was observed on post‑contrast T1‑weighted images (Figure 1D). These findings were suggestive of a necrotic lesion. As the diagnosis remained uncertain, short‑term imaging follow‑up was recommended.

**Figure 2:**
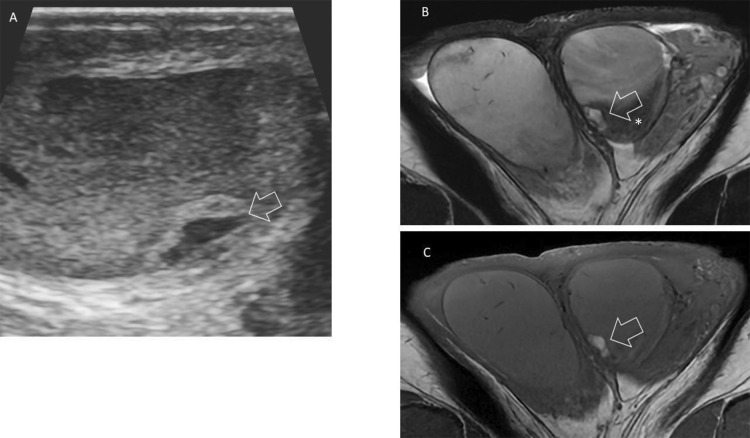
**(A)** Follow up ultrasound showed a lesion size reduction with newly appearing anechoic component on(arrow). T2 **(B)** and T1‑weighted images **(C)** showed a newly appearing hypersignal of the lesion compatible with the presence of extracellular methemoglobin (arrow) and newly hypointense area on the T2‑weighted image surrounding the lesion corresponding to segmental testicular infarction (*).

At one‑month follow‑up, ultrasound showed a reduction in lesion size with the appearance of an anechoic fluid‑filled component at the lower pole of the left testis (Figure 2A). Follow‑up MRI showed high signal intensity on both T1‑ and T2‑weighted images (Figures 2B and 2C), consistent with the presence of extracellular methemoglobin and compatible with a late subacute intratesticular hematoma associated with a segmental testicular infarction in low signal on T2‑weighted images (*).

**Figure 3:**
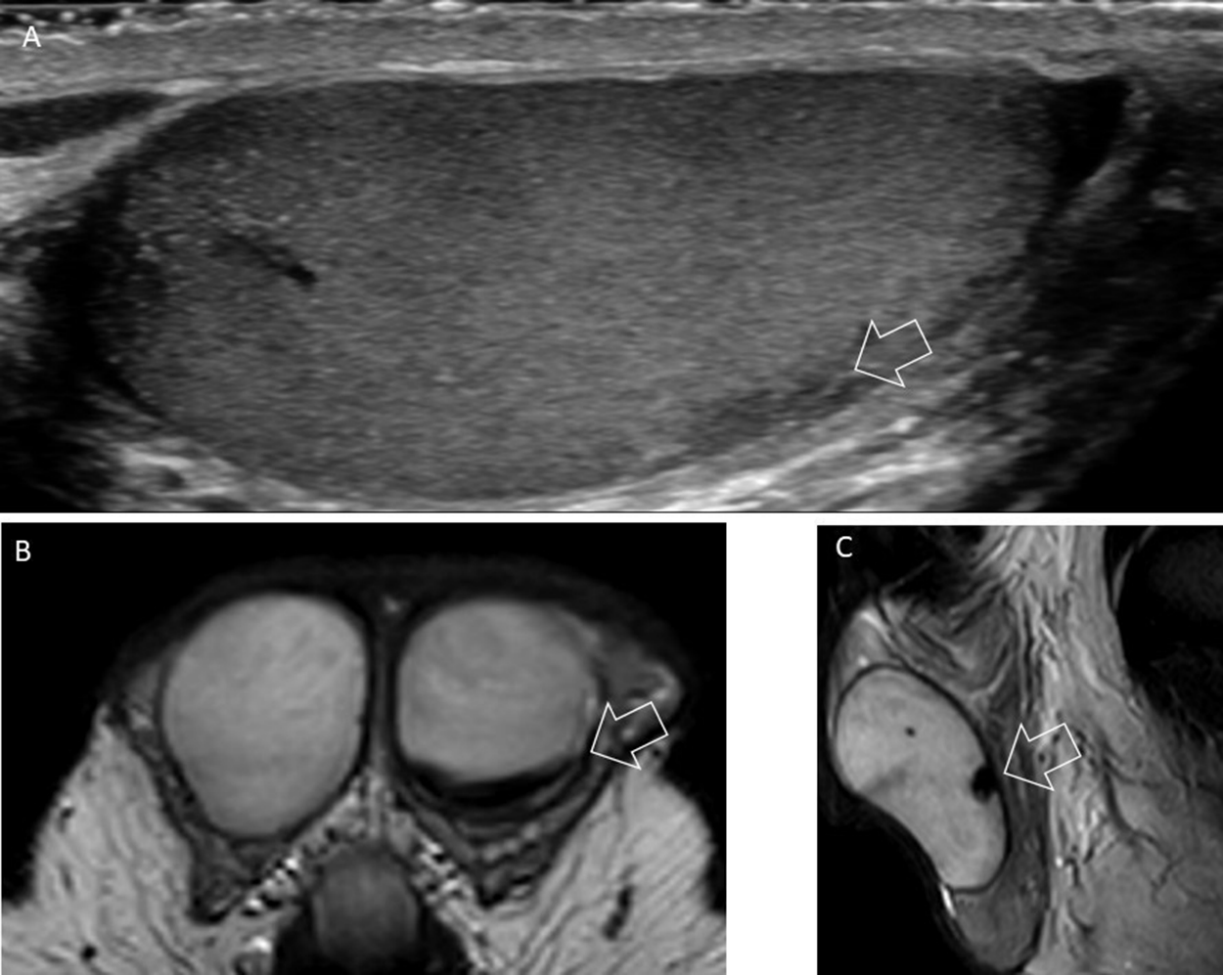
**(A)** Last follow up ultrasound showed a small residual hypoechoic area (arrow). Axial **(B)** and sagittal T2‑weighted **(C)** images showed a newly appearing hypointense signal of the lesion corresponding to hemosiderin deposition (arrow).

At six‑week follow‑up, near‑complete resolution of the lesion was observed. Ultrasound showed only a small residual mildly hypoechoic area (Figure 3A). MRI demonstrated features consistent with a chronic‑stage hematoma, including lesion regression and hemosiderin deposition, characterized by low signal intensity on axial and sagittal T2‑weighted images (Figures 3B and 3C).

## Discussion

Segmental testicular infarction is mostly idiopathic, for which no clear predisposing factors have been identified. From an ultrasonographic perspective, it represents a significant diagnostic challenge, as its imaging appearance may closely mimic that of a malignant tumor.

This diagnosis should be considered in patients presenting with acute testicular pain and scrotal swelling in the absence of trauma. It is essential to exclude coagulation disorders, predisposing factors, or unrecognized trauma; if any of these are present, this diagnosis should not be retained.

Ultrasound remains the first‑line imaging modality to exclude alternative diagnoses, such as epididymo‑orchitis, testicular torsion, or testicular neoplasm [1].

Typically, venous segmental testicular infarction appears as a solid or cystic intratesticular mass without detectable color Doppler flow corresponding to the hematoma [1].

When ultrasonography is inconclusive, MRI is useful for further lesion characterization. MRI allows identification of hemorrhagic components, the peripheral wedge‑shaped avascular area, and helps distinguish a hematoma from a solid testicular lesion by demonstrating the absence of contrast enhancement and the typical evolution of blood products over time [1].

In the absence of MRI availability or in cases of contraindication to MRI, contrast‑enhanced ultrasound may be performed to differentiate segmentation testicular infarction from a hypovascular tumor by confirming the presence of ischemic lobules (avascular lesion with perilesional enhancing rim in the subacute state).

## Conclusion

Segmental testicular infarction is a rare but important diagnostic consideration in patients presenting with acute scrotal pain and spontaneous intratesticular hematoma. Diagnosis relies heavily on imaging and careful follow‑up. The characteristic radiological evolution—progressive lesion regression and lack of enhancement – supports the benign nature of the condition and may help prevent unnecessary surgical exploration.
